# Multivalent mRNA-DTP vaccines are immunogenic and provide protection from *Bordetella pertussis* challenge in mice

**DOI:** 10.1038/s41541-024-00890-4

**Published:** 2024-06-10

**Authors:** M. Allison Wolf, Joanne M. O’Hara, Graham J. Bitzer, Elisabeth Narayanan, Dylan T. Boehm, Justin R. Bevere, Megan A. DeJong, Jesse M. Hall, Ting Y. Wong, Samantha Falcone, Cailin E. Deal, Angelene Richards, Shannon Green, Brenda Nguyen, Emily King, Clinton Ogega, Lisa Russo, Emel Sen-Kilic, Obadiah Plante, Sunny Himansu, Mariette Barbier, Andrea Carfi, F. Heath Damron

**Affiliations:** 1 Department of Microbiology, Immunology, and Cell Biology, West Virginia University, Morgantown, WV USA; 2 Vaccine Development Center at West Virginia University Health Sciences Center, Morgantown, WV USA; 3 Moderna Inc., Cambridge, MA 02139 USA

**Keywords:** RNA vaccines, RNA vaccines

## Abstract

Acellular multivalent vaccines for pertussis (DTaP and Tdap) prevent symptomatic disease and infant mortality, but immunity to *Bordetella pertussis* infection wanes significantly over time resulting in cyclic epidemics of pertussis. The messenger RNA (mRNA) vaccine platform provides an opportunity to address complex bacterial infections with an adaptable approach providing Th1-biased responses. In this study, immunogenicity and challenge models were used to evaluate the mRNA platform with multivalent vaccine formulations targeting both *B. pertussis* antigens and diphtheria and tetanus toxoids. Immunization with mRNA formulations were immunogenetic, induced antigen specific antibodies, as well as Th1 T cell responses. Upon challenge with either historical or contemporary *B. pertussis* strains, 6 and 10 valent mRNA DTP vaccine provided protection equal to that of 1/20th human doses of either DTaP or whole cell pertussis vaccines. mRNA DTP immunized mice were also protected from pertussis toxin challenge as measured by prevention of lymphocytosis and leukocytosis. Collectively these pre-clinical mouse studies illustrate the potential of the mRNA platform for multivalent bacterial pathogen vaccines.

## Introduction

*Bordetella pertussis*, the causative agent of whooping cough, is a respiratory pathogen that remains a global concern. Approximately 60 years ago, a first-generation vaccine was developed that contained formalin killed whole *B. pertussis* that was combined with diphtheria and tetanus toxoids formulated with the antigens adsorbed to alum adjuvant (DTP)^1^. Concerns regarding reactogenicity of DTP vaccines resulted in acellular pertussis vaccines being developed first in Japan, which were later formulated with diphtheria and tetanus toxoid and implemented in Europe and the United States^1,2^. Today, approximately 30 percent of the world’s population receives a pediatric acellular series of DTaP-based vaccines. In 2005, Tdap booster vaccines were made available due to rising pertussis incidence^3–5^. However, despite high vaccine coverage, transmission of *B. pertussis* remains an issue among both children and adults^6–8^. Mounting evidence supports that the humoral response elicited by these acellular pertussis vaccines wanes quickly and vaccinated individuals have the potential to serve as asymptomatic carriers which can present as a reservoir for *B. pertussis* in the population^9,10^. Additionally, it is hypothesized that vaccine pressure has resulted in strains that no longer express the pertactin antigen, which could impact vaccine efficacy^11^. In the United States, infants receive their primary series of DTaP at 2, 4, 6 months of age and because of the low number of infant deaths, it can be stated that this series is effective. However, approximately half of pertussis cases in the US are in children over one year of age and into the final DTaP boost at 4-6 years of age. It appears waning efficacy occurs during the longer interval after the primary series, and it may be possible to ameliorate this issue by replacing the Tdap booster with a superior formulation.

The original DTP vaccine has whole bacteria that was formalin killed as the antigen and thus includes approximately 2000 or more proteins with around 20 immunodominant antigens^12^. Furthermore, the DTP vaccine contains a high amount of LOS endotoxin which is a potent adjuvant on its own. Despite containing the *B. pertussis* LOS adjuvant, whole cell DTP vaccines also contain alum, in order to induce sufficient antibodies to the diphtheria and tetanus toxoids. In seminal studies performed by the Merkel laboratory, DTP immunized baboons cleared the pertussis infection more rapidly than those that were DTaP immunized^13–15^. However, only convalescent baboons, which were allowed to recover naturally from infection, were not colonized upon re-challenge^14^ and convalescent baboons also have the highest Th17 responses^14^. It is known that convalescent and DTP immunized individuals skew towards a Th1/Th17 immune response, whereas DTaP immunizations induce a more Th2 biased humoral immune response^16^. The inability of DTaP to elicit robust Th1 or Th17 immune responses nor tissue resident memory responses in mucosal tissues could be proposed for its lack of robust long-term protection^17,18^. Additionally, compared to acellular vaccines, natural infection and DTP provide more immunogens to which the host can mount protective antibodies, such as adenylate cyclase toxin (ACT) and *Bordetella* resistance to killing (BrkA), all of which have been proposed as potential vaccine candidates due to their role in disease pathogenesis^19,20^.

There is no universally agreed upon “correlate of protection” for pertussis, but higher anti-PT IgG antibody levels are associated with increased clinical protection from symptomatic disease^21^. Pertussis toxin (PT) antibodies are induced after both acellular vaccine administration and natural infection and play a role in protecting mice, baboons, and humans from clinical manifestations of disease^22,23^. DTP differs from the acellular vaccines in that it does not typically induce robust production of antibodies that recognize key secreted toxins, such as PT^24,25^. Unfortunately, antibodies to PT, produced in response to acellular vaccination, wane substantially each year post-acellular immunization in humans^26^. It has been hypothesized that epitope linked suppression due to use of chemically detoxified pertussis toxoid is also a disadvantage of the acellular vaccines^27^. A next generation *B. pertussis* vaccine would ideally stimulate both a strong cellular responses and antibodies to pertussis adhesions, toxins, and autotransporters.

Messenger RNA (mRNA) vaccines formulated in lipid nanoparticles (LNP) represent a fast, adaptable, and affordable approach to developing novel vaccines^28^. mRNA vaccines are non-infectious, non-integrating messages that can encode for immunogenic epitopes of multiple antigens^29–32^. Proteins translated in the cytosol by mRNA-LNP vaccines can be membrane bound or secreted and displayed by antigen presenting cells (APC)^29,33^. In turn, APCs can initiate a humoral and cellular immune response that results in protection from pathogen exposure^29,33^. The mRNA vaccine development process can be utilized to rapidly respond to emerging pathogens, as was displayed with the development of two mRNA vaccines for severe acute respiratory syndrome coronavirus 2 (SARS-CoV-2). FDA approved mRNA vaccines (Spikevax and Comirnaty) are were utilized to combat the SARS-CoV-2 pandemic, and both vaccines have a high seroconversion rate, protected against multiple variants, and can elicit robust B and T cell responses^34–36^. Additional immunization studies indicate that mRNA vaccines can provide protection from viruses including herpes simplex, Dengue, Zika, rabies, respiratory syncytial and cytomegalovirus^37–42^. And more recently mRNA vaccines for bacterial pathogens have been shown to have efficacy in pre-clinical studies including: *Borrelia burgdorferi* (Lyme disease), *Pseudomonas aeruginosa* (pneumonia), and *Yersinia pestis* (plague)^43–45^. A majority of the mRNA vaccine studies to date investigate mRNA vaccines as an immunization approach to protect against viral infections, but the rapid in silico design and high safety profile of mRNA vaccines suggests mRNA could be used as a strategy to prevent bacterial infections as well^29,30,35,46^. Several mRNA antigen design considerations and challenges are unique to bacterial vaccines, including the need to account for sites of unwanted mammalian glycosylation and for the potential of misfolded transmembrane domains from bacterial proteins^47^. We aimed to develop a multi-valent pertussis mRNA vaccine as a proof of principal that mRNA vaccines can be used to protect against high complexity toxigenic pathogens. An mRNA vaccine to protect against *B. pertussis* presents a unique way to increase the number of *B. pertussis* antigens, while also giving the opportunity to design a vaccine that affords protection against more than one pathogen within a combinational vaccine formulation which is preferred by both children and their parents.

Our current study aimed to design and evaluate an array of pertussis mRNA vaccines to formulate a multivalent mRNA DTP vaccine. We immunized mice to assess immunogenicity of mRNA constructs encoding key pertussis virulence factors present in the current pertussis acellular vaccines (PT, FHA, PRN, FIM), as well as antigens that are either known toxins or involved in adhesion and pathogenesis (TCFA, SHPB1, ACT). mRNA encoding diphtheria and tetanus toxoids were also added to later multivalent formulations and resulted in production of anti-diphtheria and anti-tetanus antibodies. Altogether, this collection of data demonstrates that multivalent mRNA vaccines can be immunogenic and provide protection from *B. pertussis* challenge in mice.

## Results

### mRNA transfection into mammalian cells results in expressed pertussis, diphtheria, and tetanus antigens and immunogenicity in mice

**Fig. 1:**
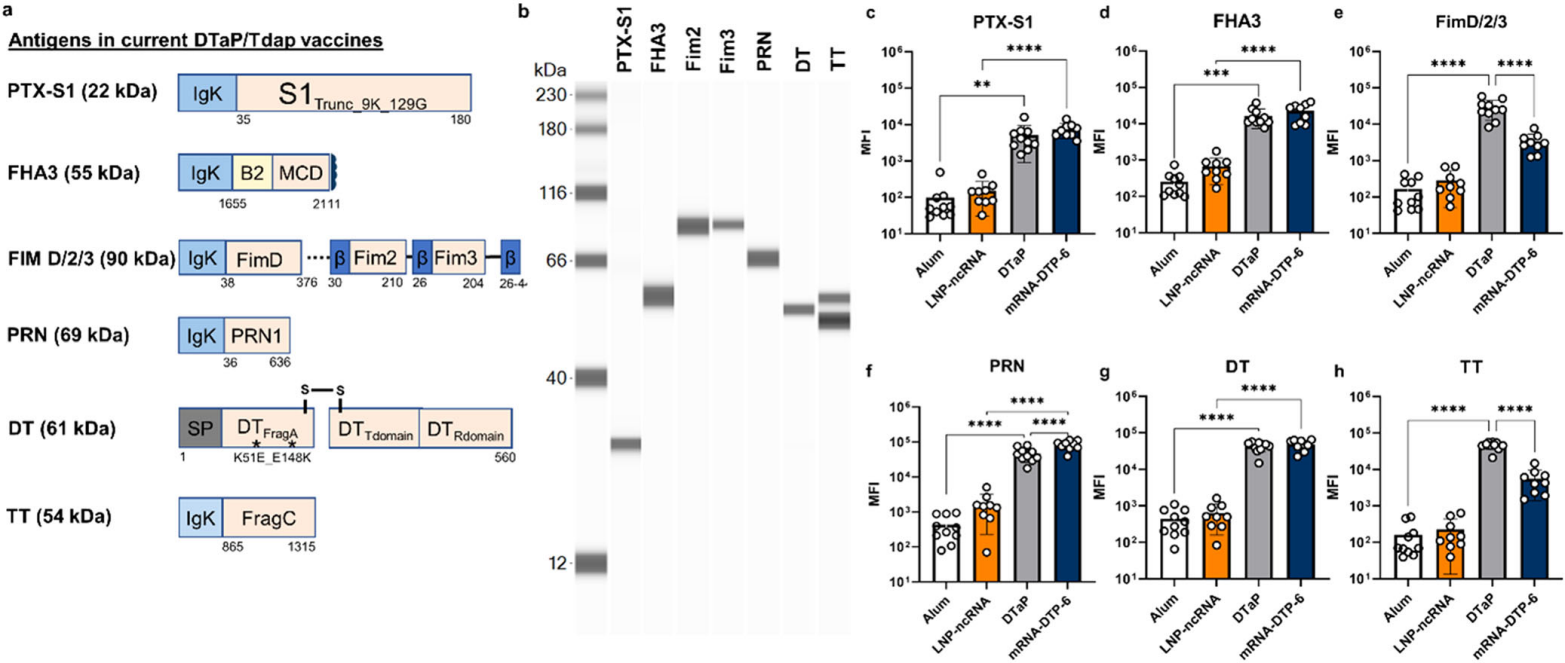
Bacterial mRNA constructs are translated into protein in mammalian cells in vitro and are immunogenic in mice. **a** Schematic of mRNA design for each antigen, describing (1) the protein sequence, (2) the signal sequence and (3) toxin-inactivating mutations included in the construct. The expected molecular weight of the translated protein is included in parentheses. **b** Following transfection of Expi293F cells with individual mRNA constructs, supernatants were collected 48 h later and evaluated for detection of secreted pertussis, tetanus and diphtheria proteins using the JESS system and antigen specific antibodies. **c**–**h** Sera collected from 8-week-old C57BL/6 J mice one-month post-boost were evaluated for antigen specific antibodies using a Luminex assay which has beads coated with native antigens purified from (**d**–**f**) *Bordetella pertussis* (FHA, FIM 2/3, PRN) and (**c**, **g**, **h**) detoxified PT, DT, and TT toxoids. The mRNA-DTP-6 vaccine was able to induce *Bp* antigen specific antibodies comparable to DTaP vaccination. Bar graphs are mean with error bars shown as ±SD. A two-way ANOVA with Tukey’s post-hoc test was used to calculate significant difference. **p < 0.0021, ****p < 0.0001. LNP-ncRNA= Lipid Nanoparticle- noncoding RNA; mRNA-DTP-6= mRNA- Diphtheria Tetanus Pertussis 6 antigen.

All DTaP vaccines contain pertussis toxoid (PT), filamentous hemagglutinin (FHA), pertactin (PRN), diphtheria toxoid (DT) and tetanus toxoid antigens (TT) and several commercial formulations contain fimbriae 2/3 adhesion as well (*e.g*. Daptacel, Sanofi). To develop mRNA constructs of each DTaP antigen, IgK or native signal peptides were used as secretion sequences for each antigen (Fig. 1a). Based on optimized antigens from the literature and predicted effects on protein expression, truncations of each antigen were designed. For pertussis toxoid antigen, 22 kDa truncated PtxA protein sequence (S1 subunit) was utilized^48^(Fig. 1a). The FHA antigen was based on the Mal85 or Mature C terminal Domain of FHA as antibodies generated to the beta barrel stalk of FHA have little to no function^49,91^. In order to induce antibodies to all of the serotypes of fimbriae, a fusion of FimD/Fim2/Fim3 was developed (Fig. 1a). Despite many strains not expressing the PRN antigen, it was still included because many circulating strains in Europe still express PRN^50^. And finally, DT and TT antigens were designed based on their known fragments that induce protective antibodies (Fig. 1a). To confirm that the mRNA designs lead to expression of each antigen, 15 ug of each mRNA were transfected into Expi293F cells and a Jess system was utilized to detect secreted protein. All mRNAs evaluated resulted in expression of proteins at their expected molecular weights (Fig. 1b). Of note, the TT antigen shows two different bands which suggests there may be a N-glycosylation site within the antigen that has not previously been reported. To investigate and compare the immunogenicity of an mRNA-DTP vaccine to acellular protein DTaP vaccine (1/20th human dose), mice were immunized with Alum, DTaP (Daptacel, Sanofi), mRNA-DTP-6 vaccine (10 µg), or a control mRNA formulated vaccine containing a non-coding mRNA (LNP-ncRNA). Luminex analysis of antibodies produced by mice immunized with mRNA-DTP-6 vaccine confirmed that all of the antigens were immunogenic (Fig. 1c–h, Sup Fig. 1). In relation to DTaP, the mRNA vaccine induced comparable levels of anti-FHA, PTX-S1, and DT toxoid antibodies (Fig. 1c, d, g). There was also a significant increase in the level of anti-PRN antibodies from mRNA-DTP-6 vaccination compared to DTaP vaccination (Fig. 1f). Finally, although there was a significant decrease in anti-FimD/2/3 and TT specific antibodies from mRNA-DTP-6 vaccination compared to DTaP vaccination these antigen specific antibodies were present and roughly two logs higher than the negative control (Fig. 1e, h). Overall, this data suggests that mRNA-DTP-6 vaccine can elicit antigen specific responses comparable to the current DTaP protein vaccine in mice.

### mRNA-DTP-6 elicits a splenic memory T cell response after boost in C57BL/6 mice

**Fig. 2:**
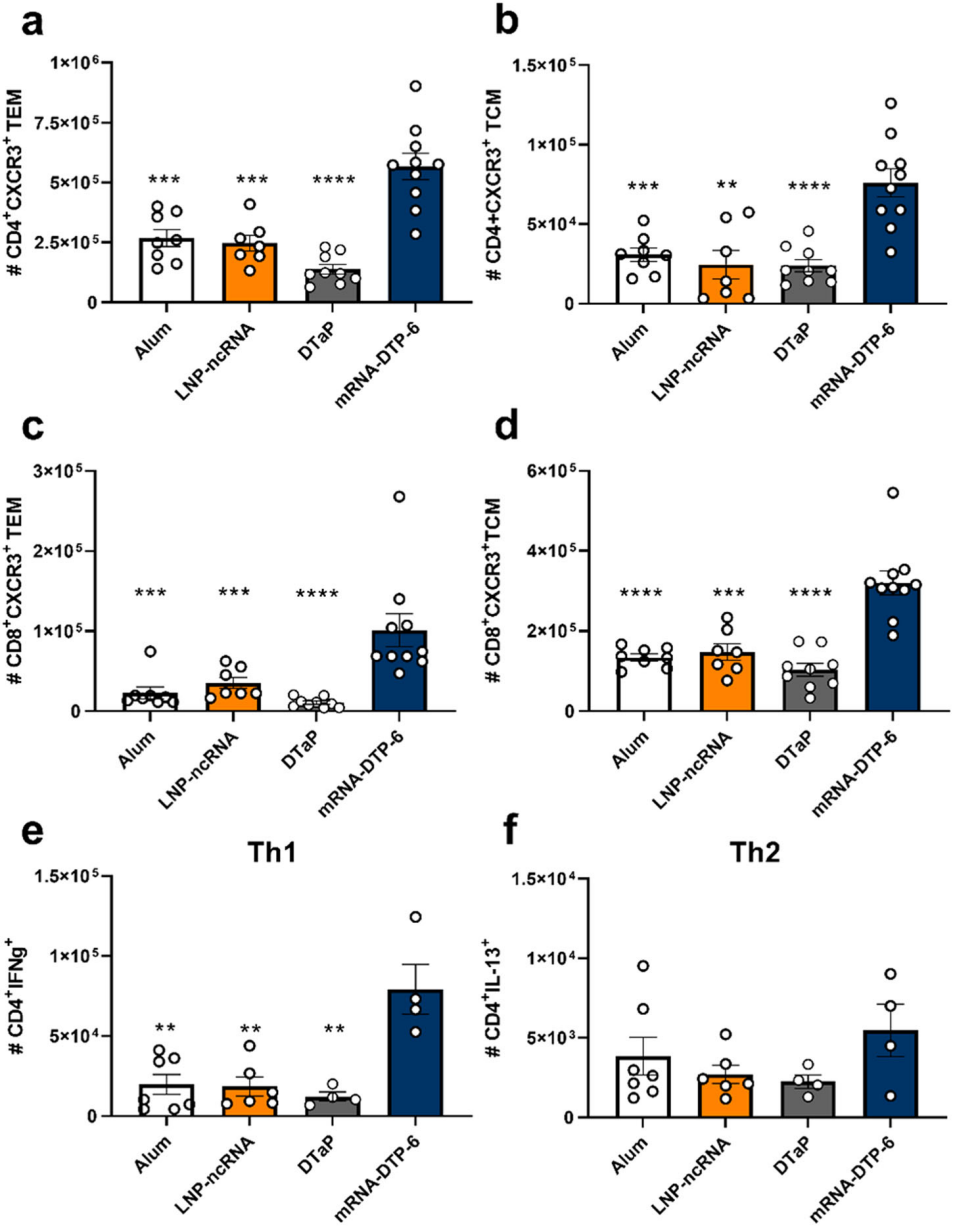
mRNA-DTP-6 vaccination induces more splenic CD4 and CD8 memory T cells than DTaP vaccination. Flow cytometry was used to measure Splenic memory T cells. mRNA-DTP-6 elicited statistically higher levels of CD4^+^CXCR3^+^ TEM (**a**) and TCM (**b**) and CD8^+^CXCR3^+^ TEM (**c**) and TCM (**d**) than DTaP vaccinated mice. T cell cytokine responses were also measured with flow cytometry and mRNA-DTP-6 induced higher levels of CD4^+^IFNγ^+^ (**e**) which suggest a more Th1-skewed cell mediated response than DTaP vaccinated mice. There was no statistical difference between mRNA-DTP-6 and DTaP CD4^+^IL-13^+^ T cells (**f**). Error bars are mean ± SEM. A Mann–Whitney two-tailed with 95% confidence interval test was used to determine statistical difference from mRNA-DTP-6; **p < 0.0021, ***p < 0.0002, ****p < 0.0001. LNP-ncRNA= Li*p*id nanoparticle- noncoding mRNA; mRNA-DTP-6= mRNA- Diphtheria, Tetanus, and Pertussis 6 antigen vaccine.

Durability of protection and T cell responses are important aspects of acellular pertussis vaccines that warrant improvement in next generation formulations. Therefore, we next investigated the splenic memory T cell responses in the same mice by flow cytometry and found that the mRNA-DTP-6 vaccine elicits significantly more CD4+ effector memory T cells (TEM) and CD4+ central memory T cells (TCM) than DTaP (Fig. 2a, b, Supplementary Fig. 3). Interestingly we also observed higher numbers of CD8+ TEM and TCM in mice immunized with mRNA-DTP-6 compared to mice immunized with DTaP (Fig. 2c, d). Furthermore, when T cell cytokine responses were explored, we observed that the mRNA-DTP-6 vaccine elicited significantly higher levels of IFNg+ CD4+ T cells than DTaP, as well as the LNP-ncRNA control group (Fig. 2e, Supplementary Fig. 3). Th2 responses were measured by CD4 + IL-13+ cells and responses between DTaP and mRNA-DTP-6 were similar and not significantly different (Fig. 2f, Supplementary Fig. 3). These data support that the mRNA-DTP-6 can induce robust Th1 and CD4 IFN+ responses in contrast to DTaP.

### Immunization with mRNA pertussis vaccines elicits antibodies that bind *B. pertussis* and pertussis toxin

**Fig. 3:**
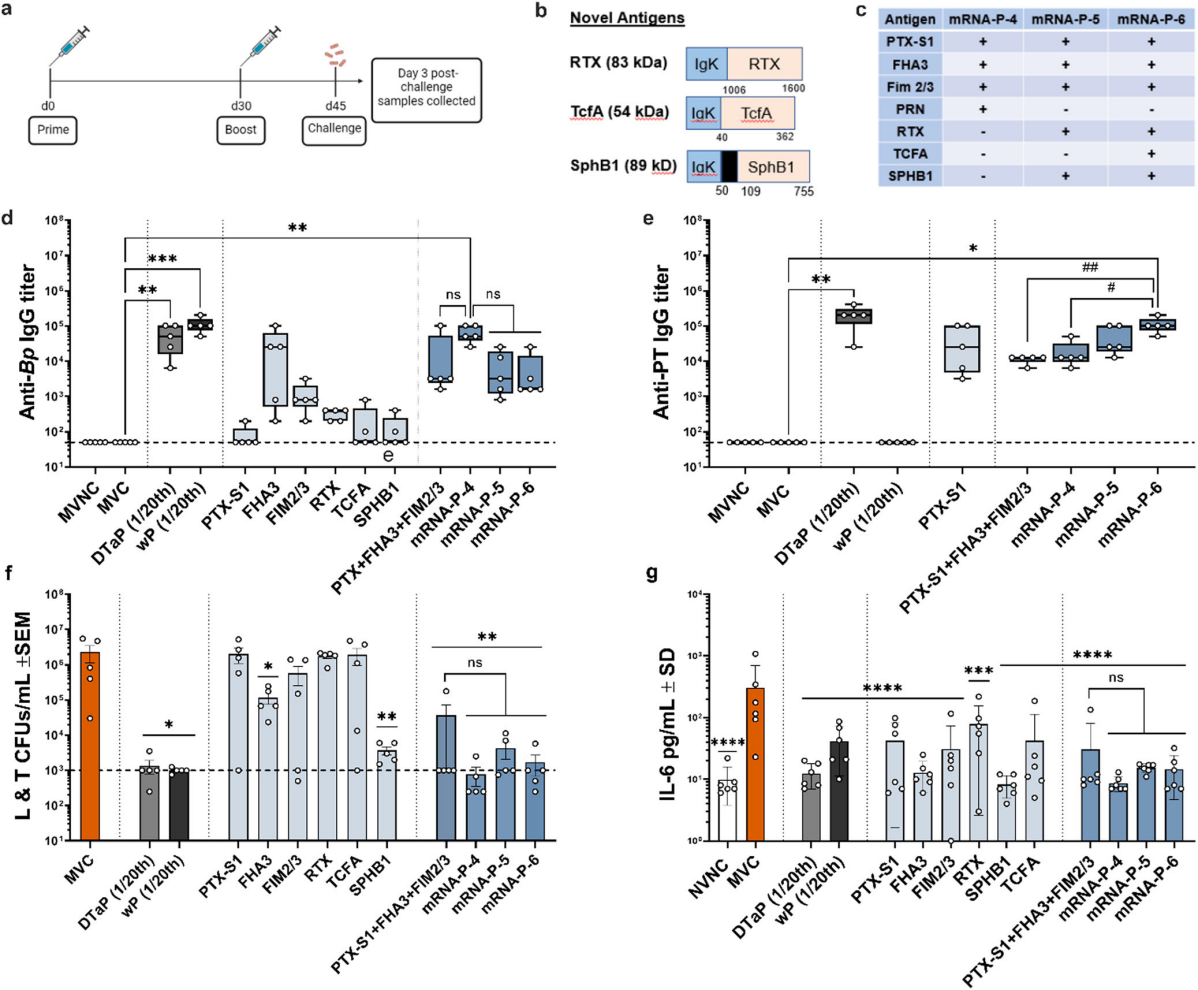
IgG antibody titers in the serum, bacterial burden in the respiratory tract, and pulmonary IL-6 production 3 days after *B. pertussis* isolate UT25Sm1 challenge using a C57BL/6 J model. **a** Vaccination and challenge model utilized to evaluate mRNA vaccine immunogenicity and protection. **b** Schematic on novel mRNA constructs included in mRNA vaccination. **c** A chart listing greater combination of mRNA and antigens included. Serum IgG antibody titers to *B. pertussis* (*Bp*) (**d**) and PT (**e**) were determined by ELISA assay. *B. pertussis* was quantified by counting serially diluted CFUs following challenge with isolate UT25Sm1. CFU counts were determined from lung and trachea (L &T) homogenate (**f**). **g** Pro-inflammatory cytokine, IL-6, was quantified in the lungs at 3 days post-challenge using a V-Plex cytokine kit. Kruskal-Wallis with Dunn’s post-hoc test (**d** and **e**) or a one-way ANOVA with a Tukey’s post-hoc test (**f**, **g**) were used to calculate differences to MVC; *p < 0.05, **p < 0.005, ***p < 0.005. Another Kruskal-Wallis with Dunn’s post-hoc test (d and e) or a one-way ANOVA with a Tuk**e**y’s post-hoc test (f and g) were used to calculate di**f**ferences between multivalent vaccine formulation; ^#^*p* < 0.05, ^##^*p* < 0.005. Box and whisker plots display minimum to maximum values with all data points. MVNC = mock-vaccinated and non-challenged; MVC = mock-vaccinated and challenged; SEM = standard error of the mean; SD = standard deviation; ns= non-significant, *p* > 0.05. *n* = 5 per treatment group. Dotted lines indicate lowest limit of detection. Consult Table 1 for vaccine antigen description and Table 2 for vaccine name.

To evaluate and compare the immunogenicity of additional pertussis antigens, as single antigen vaccines or included in multivalent combination vaccines, mice were immunized with individual antigens RTX, TCFA, and SphB1 (Table 1, Fig. 3b), or in combination with pertussis antigens in the current DTaP and Tdap vaccines (Table 1, Fig. 3c). Single and combination antigen mRNA vaccine responses were compared to 1/20th human dose DTaP or 1/20th human wP vaccine (lacking DT and TT antigens). The majority of mRNA pertussis vaccines containing single antigens induced low levels of antibodies that bind *B. pertussis* bacterium, however when mRNA antigens were combined, anti- *B. pertussis* titers were increased, and titers for mRNA-P-4 vaccine were significantly higher than those for the MVC group and no statistical difference was measured against other multivalent formulations (Fig. 3d). Furthermore, these anti- *B. pertussis* antibodies were comparable to levels elicited by DTaP and wP immunized mice (Fig. 3d). Mice immunized with the PTX-S1 mRNA vaccine induced similar levels of anti-PTX antibodies as animals immunized with DTaP. Furthermore, all combination mRNA vaccines containing PTX-S1 mRNA induced detectable anti-PT IgG antibodies in the serum although mRNA-P-6 induced statistically significant higher titers than the PTx-S1 + FHA3 + FIM2/3 and mRNA-P-4 formulations (Fig. 3e). Based on these data, we can conclude that the PTX-S1 antigen consisting of genetically detoxified S1 subunit (C180) is sufficient to induce anti-PT antibodies that recognize PT holotoxin^48^. Furthermore, antibody interference was not observed when up to 6 antigens were combined into the formulation.

**Table 1:** Description of antigens selected for mRNA vaccines

mRNA antigen acronym	Description of mRNA construct	Role in pathogenesis
PTX-S1	Consists of the PTX S1 subunit of *Bp* Tohama I strain, truncated at residue 180 to remove the hydrophobic C-terminal tail and increase expression^90^. The native signal peptide was removed, and the construct was detoxified by mutating residue 9 from an arginine to lysine and residue 129 from a glutamic acid to a glycine^48^.	PT is a secreted exotoxin involved in the colonization and respiratory tract and induces leuko- and lymphocytosis
FHA3	Encodes filamentous hemagglutinin representing residues 1655-2111 of full-length Tohama I hemagglutinin. This fragment was previously published as MAL85^91^.	Cell surface or secreted protein that mediates adherence to epithelial and immune cells
FIMD/2/3	Encodes a mammalian mimetic containing segments of both fimbrae-2 and fimbrae-3. Consists of FimD tip protein with its native signal peptide removed (residues 38-376), a GGGS linker, the Fim2 protein with its native signal peptide removed (residues 30-210) a second GGGSGGGS linker, the Fim3 protein with its native signal peptide removed (residues 26-204), a third GGGS linker, and then amino acids 26-44 of Fim3. Five mutations were introduced to avoid putative N-linked glycosylation sites.	Role in attachment to respiratory epithelial cells
PRN	Consists of the passenger domain of pertactin (residues 35-636) of the Tohama I strain. One mutation was introduced to avoid a putative N-linked glycosylation site.	Adhesin involved in attachment to epithelial cells, specifically the trachea epithelial cells
DT	Consists of the full-length diphtheria toxin from *Corynebacterium diphtheriae*, with two mutations (K51E/E148K) to eliminate its enzymatic activity. Three N-glycosylation modifications were introduced to avoid N-glycosylation events.	Essential toxin for *Corynebacterium diphtheriae* which kills cells by inhibiting protein synthesis
TT	Consists of fragment C receptor domain from tetanus toxin from *Clostridium tetani*. Five N-glycosylation modifications were introduced to avoid N-glycosylation events.	Essential toxin for *Clostridium tetani* and inhibits nerve terminals leading to muscle rigidity and spasm
RTX	Consists of residues 1006 to 1600 of the C-terminal repeats in toxin domain (RTX) of adenylate cyclase toxin (ACT) from *Bp* Tohama I strain. Four mutations were introduced to avoid putative N-linked glycosylation sites.	ACT is a secreted toxin that binds to phagocytic cells and generates cAMP upon activation, which leads to cellular intoxication
TCFA	Consists of the passenger domain of the *Bp* Tohama I strain tracheal colonization factor (residues 40-362).	TCFA is an autotransporter and in a murine model, deletion of this protein decreased colonization
SPHB1	Consists of the passenger domain of subtilisin-like protease B1 from the Tohama I strain (residues 50-752).	Cleaves FhaB to generate FHA
BRKA	Consists of the passenger domain of BrkA (residues 42-732) of the Tohama I strain. One mutation was introduced to avoid a putative N-linked glycosylation site.	Inhibits complement activation

### Single and multivalent antigen mRNA DTP vaccines provide protection and decrease bacterial burden post *B. pertussis* challenge

Next, we evaluated and compared the efficacy of the individual antigen and combination antigen mRNA vaccines to 1/20th the human dose^51,52^ of either DTaP or wP (Fig. 3f, g) in a murine *B. pertussis* challenge model. At day 3 post challenge with *B. pertussis* isolate UT25Sm1, bacterial burden was determined in the trachea and lungs of mice. As expected, since PT is secreted from the bacterium, mice immunized with PTX-S1 did not have decreased bacterial burden, (Fig. 3f). Conversely, mice immunized with FHA3 had a significant decrease in bacterial burden whereas FIM2/3, TCFA and RTX immunized mice did not have a significant decrease in bacterial burden (Fig. 3f). Interestingly, mice immunized with the SPHB1 single antigen, had significantly reduced bacterial burden, despite barely detectable anti-BP IgG titers (Fig. 3d, f). mRNA vaccines that combined 3 or more antigens decreased the bacteria burden in lung and trachea at day 3 post-challenge compared to the MVC group (Fig. 3f). Furthermore, the multivalent mRNA vaccines elicited protection comparable to DTaP and wP in the lung and trachea (Fig. 3f). Pro-inflammatory cytokines, including interleukin-6 (IL-6), are known to be significantly elevated during a *B. pertussis* infection in mice, baboons, and humans^53^. Interestingly, all the vaccines, regardless of the bacterial burden after challenge had decreased production of IL-6 in the lung at day 3 post-challenge when compared to the MVC group (Fig. 3g). In summary we observed that multivalent mRNA vaccines stimulated the production of both anti-*B. pertussis* and anti-PT IgG antibodies, decreased respiratory bacterial burden, and prevented high IL-6 production in the lungs compared to the MVC group and these endpoint values were similar to DTaP immunized mice (Fig. 3d–g). Overall, these data show that mRNA vaccines can provide protection against *B. pertussis* challenge in mice.

### Immunization with mRNA-DTP-10 vaccine elicits a balanced Th1/Th2 antibody response similar to DTP

**Fig. 4:**
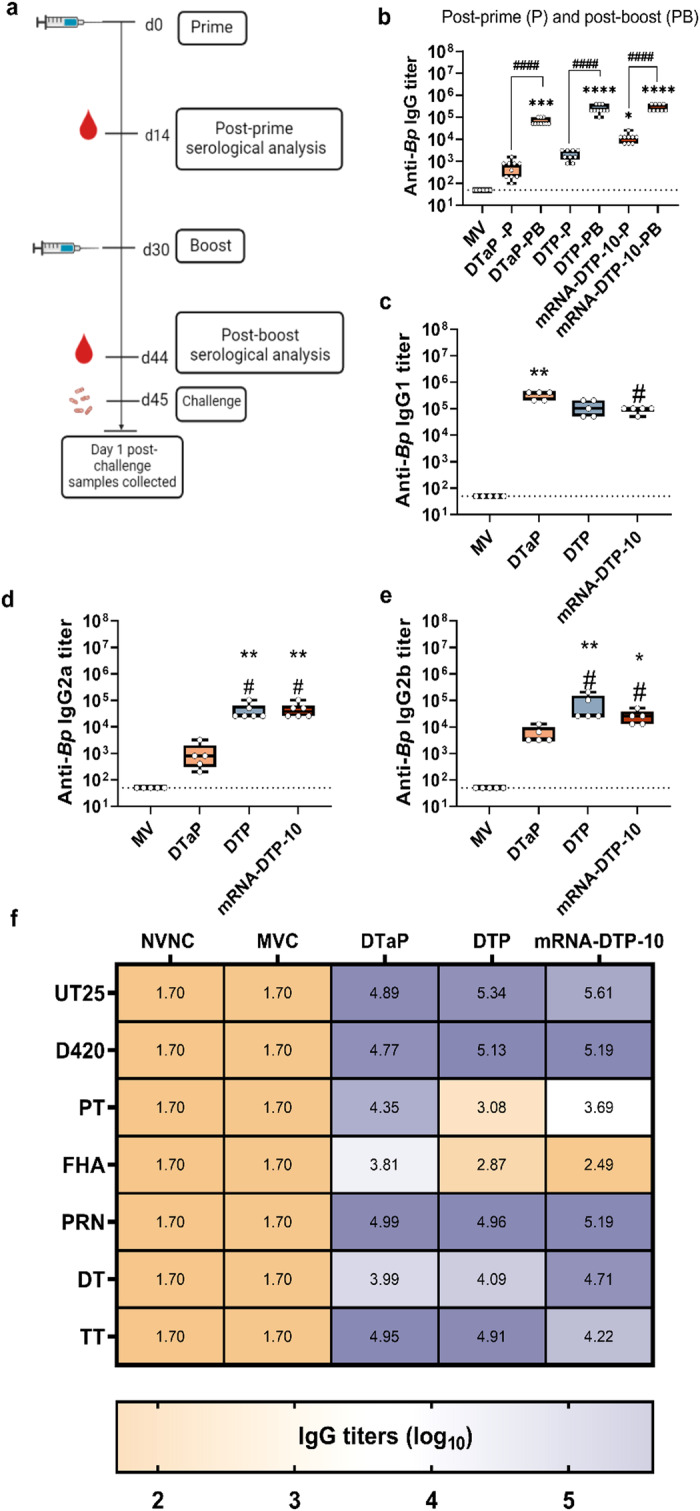
Immunological response quantified post-prime and post-boost. **a** Immunization and serum collection timeline. **b** Serum IgG antibody titers to *B. pertussis* (*Bp*) strain UT25 were analyzed by ELISA assay two weeks after the prime and boost immunization. Post-boost Th1/Th2 IgG antibodies in the sera quantified via ELSA assay. Post-boost anti-*B. pertussis* (*Bp*) IgG1 (**c**), IgG2a (**d**), IgG2b (**e**) response after boost. **f** Heat map depicting serological IgG titers recognizing pertussis, diphtheriae, and tetanus antigens 1-day post-challenge in BALB/c mice. IgG antibodies to *B. pertussis* strains UT25 and D420, PT, FHA, PRN, RTX, DT, and TT depicted in log scale. MVMC= mock-vaccinated, mock-challenged; MVC=mock-vaccinated and challenged; n = 10 per treatment group, except for MVMC *n* = 5. Kruskal–Wallis with Dunn’s post-hoc test was used to calculate differences to MVC; *p < 0.05, ***p < 0.005, ****p < 0.001 indicate differences from MV and #*p* < 0.05 indicates differences from DTaP. A Student’s *t* test was performed to compare differences in IgG titers between prime and boost for each vaccine. Box and whisker plots display minimum to maximum values with all data points. MV=mock-vaccinated; SD= standard deviation. *n* = 5–6 per treatment group. Dotted lines indicate lowest limit of detection.

To investigate the impact of an additional pertussis antigens to the B. pertussis vaccine induced antibody response, we evaluated a 10-antigen mRNA-DTP-10 formulation containing all of the current acellular pertussis, RTX, TCFA, SpHB1, BrkA, and DT/TT antigen constructs (Table 1). We added an mRNA for BrkA as it has value as a pertussis antigen (Tables 1, 2). mRNA-DTP-10 (10 µg) was compared to acellular (DTaP) and whole cell pertussis vaccines (Serum Institute of India Triple Antigen, DTP)). Mice were primed and boosted four weeks later with the same formulations. Serological analysis was performed at two weeks post prime, to weeks post boost and at day one post challenge (Fig. 4a). DTaP immunized mice had the lowest level of antibodies binding whole *B. pertussis* bacterium at post prime. DTaP, DTP, and mRNA-DTP-10 antibody levels were comparable at post-boost (Fig. 4b). We next evaluated the isotypes of *B. pertussis* specific IgG antibodies produced by each vaccine (Fig. 4c–e). DTaP immunized mice had the highest amount of IgG1 antibodies which is indicative of Th2 responses (Fig. 4c). Both DTP and mRNA-DTP-10 vaccines induced significantly higher amounts of IgG2a and IgG2b antibodies than DTaP (Fig. 4d, e). This data further supports the T cell immunogenicity data (Fig. 2) that mRNA-DTP-10 can elicit a more robust Th1 response, in contrast to DTaP.

**Table 2:** Nomenclature of multivalent pertussis (P) and diphtheriae (D), tetanus (T) mRNA

Vaccine name	Included antigens
mRNA-P-4	PTX-S1, PRN, FHA3, FIM2/3
mRNA-P-5	PTX-S1, FHA3, FIM2/3, RTX, SPHB1
mRNA-P-6	PTX-S1, FHA3, FIM 2/3, RTX, SPHB1, TCFA
mRNA-DTP-10	PTX -S1, PRN, FHA3, FIM2/3, RTX, BRKA, TCFA, SPHB1, DIP, TET

### mRNA-DTP-10 elicits broad serological response to *B. pertussis* bacterium as well as pertussis, diphtheria, and tetanus antigens

Serum antibodies at one day post challenge were used to determine antigen specific binding to whole *B. pertussis* bacteria (UT25 or D420), PT, FHA, PRN, DT, and TT. DTP and mRNA-DTP-10 induced the highest amounts of antibodies to whole bacterium compared to DTaP (Sup Fig. 2). Antibodies that recognize PT were highest in DTaP immunized mice (Fig. 4f); however, that is to be expected since DTaP contains PT holotoxin as opposed to PTX-S1 (mRNA). Furthermore, DTP whole cell vaccines are also known to induce very low levels of antibodies to PT^54^. DTaP also induced the highest amount of FHA antibodies (Fig. 4f). mRNA-DTP-10 contains the truncated MCD FHA antigen, and these ELISAs were performed with full length FHA protein antigen from *B. pertussis*, and this difference is likely due to the utilization of the smaller MCD like FHA antigen construct in mRNA-DTP-10. DTaP, DTP, and mRNA-DTP-10 immunized mice have comparable levels of antibodies to PRN, DT, and TT antigens (Fig. 4f). Overall, these data suggest that the multi-valent mRNA-DTP-10 is able to induce robust antibodies specific for the antigens of interest even in a combinational 10 antigen formulation.

### mRNA-DTP-10 provides protection and facilitates clearance of both classical UT25 (1977) and contemporary D420 (2002) *B. pertussis* strains

**Fig. 5:**
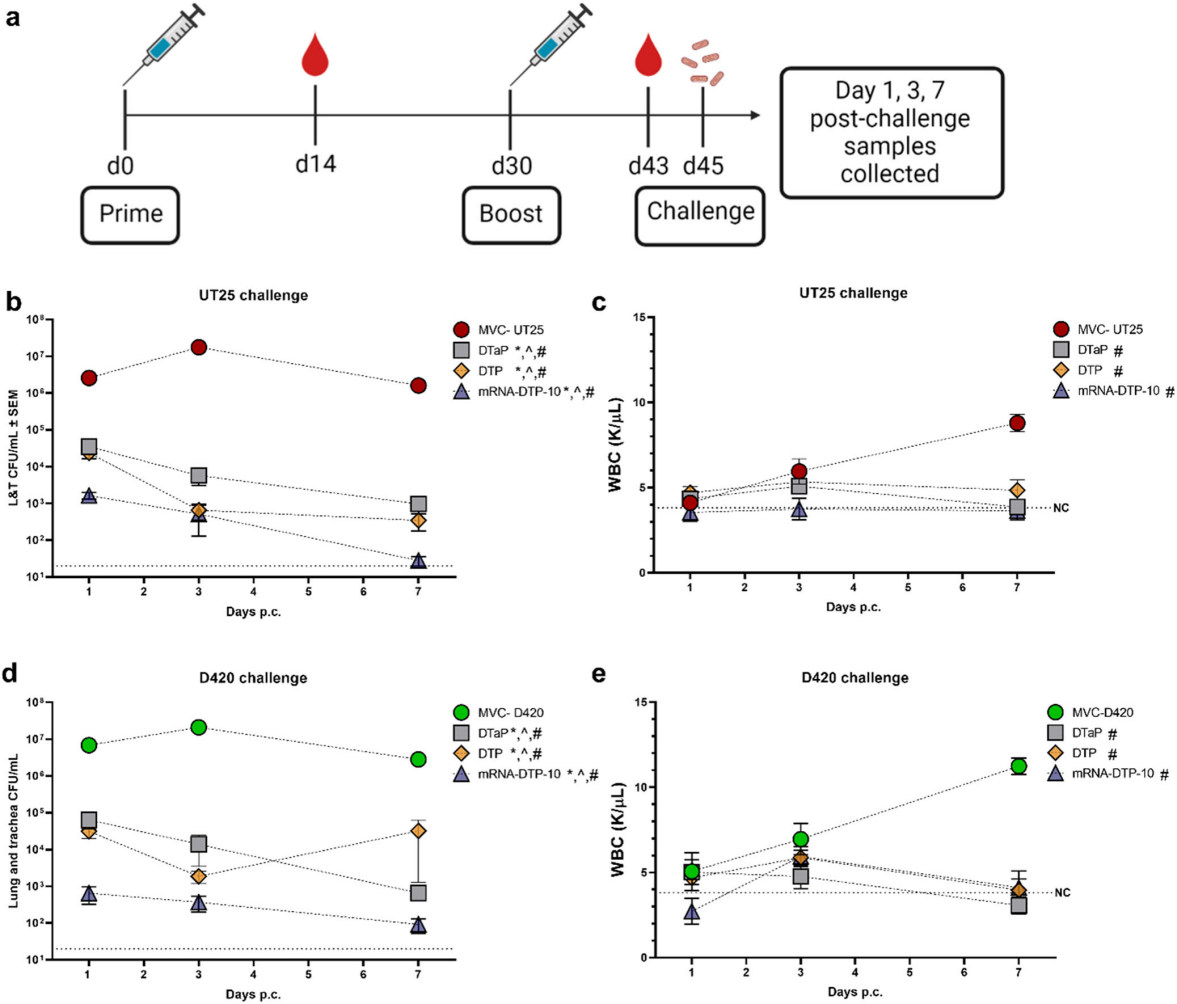
Bacterial burden and white blood cell count after challenge with two different *B. pertussis* strains. Bacterial burden and white blood cell count (WBC) were enumerated after challenge with two strains of *B. pertussis* in BALB/c mice at 3 time points. **a** Timeline for vaccination, bacterial challenge, and end-point assays. **b**, **d** CFU’s were calculated at days 1, 3, and 7 post-challenge from the lung and trachea homogenates of mice challenged with *Bp* UT25 (**b**) and D420 (**d**). **c** and **e** WBC counts were quantified in blood at days 1, 3 and 7 post-UT25 (**c**) and D420 challenge (**e**) using an IDEXX Procyte. **b**, **d** Dotted line indicates lowest limit of CFU detection, while dotted line in (**c**) and (**e**) indicates the average WBC count of non-challenged mice. A one-way ANOVA with Tukey’s post-hoc test calculated differences to MVC. *indicates a significant difference from MVC at day 1, ^ indicates difference from MVC at day 3, and # indicates a difference from MVC at day 7. MVC = mock-vaccinated and challenged; SEM=standard error of the mean. *n* = 5 per treatment group. Consult Table 1 for vaccine antigen description and Table 2 for vaccine composition.

In most studies, classical lab passaged strains such as Tohama I, BP338, UT25 are used for murine challenge studies. Vaccine pressure has played a role in the genome evolution of *B. pertussis* in the past 30 years^55^. The CDC Pertussis laboratory has performed extensive genomic analysis and grouped contemporary strains into clades^55,56^. The Merkel lab and others have used the D420 strain in the baboon model and we have used it in two rat challenge studies as well^57–59^. D420 belongs to the clade 013 which is a major clade observed in the most recent genomic surveys. D420 was selected due to its continued use in the baboon model. One caveat to D420 is that unlike many recent *B. pertussis* strains, it does have a wild type pertactin gene and expresses the protein antigen. In our studies we included pertactin antigen and therefor D420 was used an appropriate challenge strain given the goals of the study. Due to the accumulating amount of challenge data with D420, we selected it as a representative challenge strain. Mice were primed and boosted with the same vaccines described above (Fig. 5a) and challenged with either UT25 or D420. We determined bacterial burden and white blood cell counts at days 1, 3, and 7 post challenge (Fig. 5b–e). mRNA-DTP-10 immunized mice were observed to have the highest reduction of bacterial burden in both UT25 or D420 challenged mice (Fig. 5b, d). White blood cell counts typically raise over time post *B. pertussis* challenge in non-immune mice. DTaP, DTP, and mRNA-DTP-10 all suppressed leukocytosis/lymphocytosis (Fig. 5c, e), suggesting that antibodies to pertussis toxin were able to neutralize the toxin and prevent harmful effects.

### mRNA-DTP-10 prevent lympho- and leukocytosis after PT challenge

**Fig. 6:**
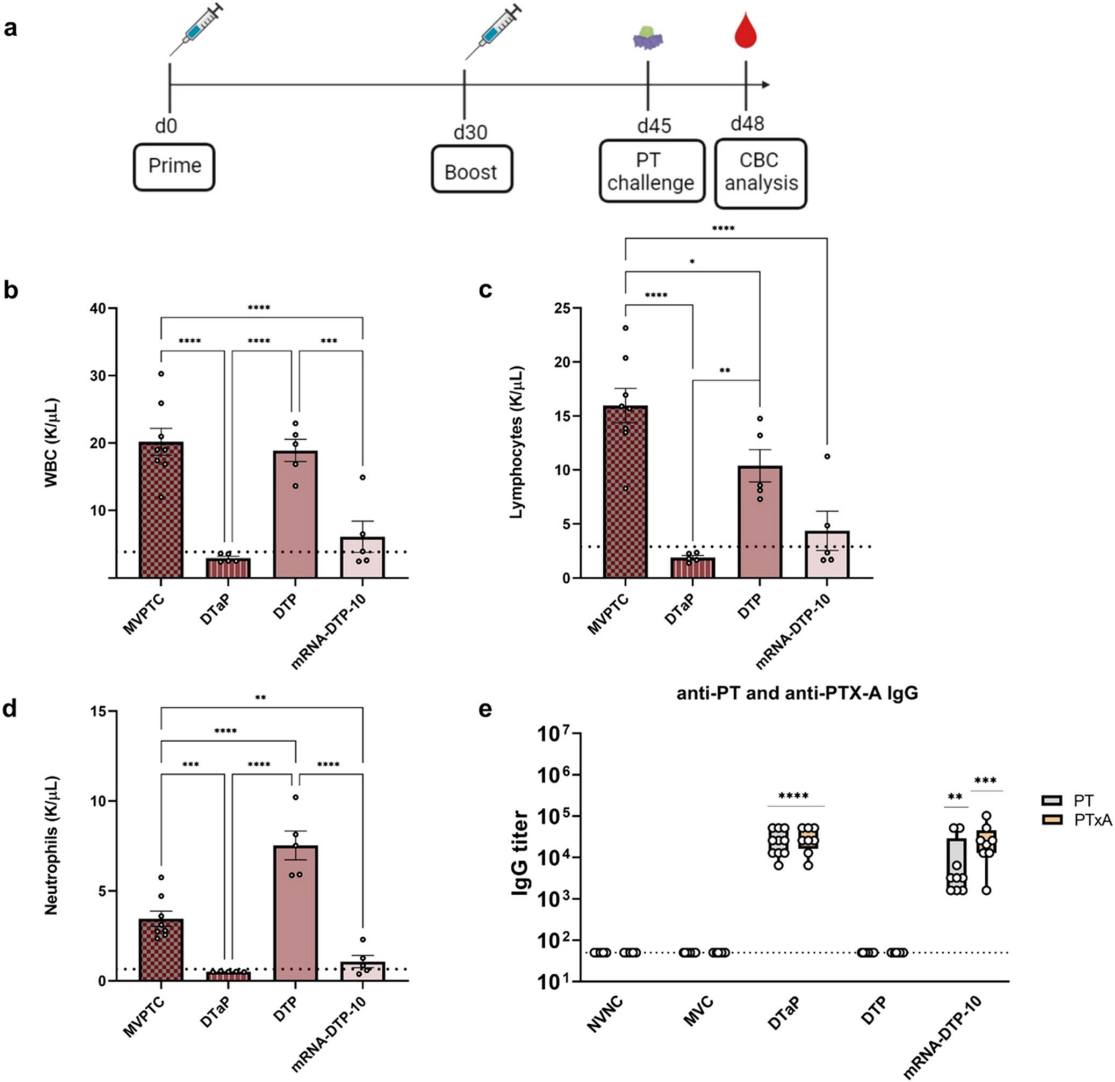
Complete blood cell counts and anti-PT IgG titers in mice after PT challenge. **a** Schematic of vaccination and PT (2 µg) challenge using BALB/c mice. **b** White blood cell (WBC), lymphocyte (**c**) and neutrophil (**d**) counts were quantified in blood 3 days post-PT challenge using an IDEXX Procyte. **e** Post-boost sera were analyzed for IgG antibody titers to holotoxin PT and PTx-S1 antibodies by ELISA. Dotted line in panels (**b-d**) depicts average WBC, neutrophil and lymphocyte counts in the blood for mock-vaccinated and non-challenged mice and in panel (**e**) dotted line depicts lower limit of detection. Box and whisker plots display minimum to maximum values with all data points. A one-way ANOVA with Tukey’s post-hoc test calculated differences in (**b**–**d**) (*p < 0.05, **p < 0.01, ***p < 0.005, ****p < 0.001). Kruskal-Wallis with Dunn’s post-hoc test was used to calculate differences to MVC in panel (e); **p < 0.01, ***p < 0.005, ****p < 0.001 indicate differences from MV MVPTC = mock-vaccinated and PT challenged. Consult Table 1 for vaccine antigen description and Table 2 for vaccine composition.

PT contributes to the pathogenesis of *B. pertussis* by inducing leukocytosis, hypoglycemia, and histamine sensitization. Due to its important role in pathogenesis, some countries have elected to use a mono-valent PT vaccines^5,60–64^. Further highlighting the importance of PT neutralization, a humanized anti-PT antibody prevented disease severity in baboons^23,65^. These examples support the importance of PT neutralization. The mRNA-DTP-10 formulation contains an mRNA construct encoding for a portion of the detoxified A subunit of PT (Tables 1 and 2). mRNA-DTP-10 induced levels of anti-PT IgG antibodies comparable to DTaP (Fig. 4f) and to evaluate the function of these anti-PT antibodies, a mouse-PT challenge model was utilized (Fig. 6a). Immunization with mRNA-DTP-10 prevented increased white blood cell, neutrophil, and lymphocyte counts 3 days after challenge with 2 µg of PT (Fig. 6b–d). The reduction of these immune cells was comparable to DTaP, which has detoxified PT containing all the subunits (Fig. 6b–d). Of additional note, while the DTP vaccine prevents bacterial burden (Fig. 5b, d), it did not prevent leuko- or lymphocytosis in the PT challenge model (Fig. 6b–d). Lastly, we measured anti-PtxA (S1) subunit or anti-PT holotoxin IgG titers. mRNA-DTP-10 vaccine only contains a mRNA for C180 of the S1 subunit. Interestingly, DTaP immunization appears to induce a similar amount of antibodies to both S1 or holotoxin, suggesting to us that most antibodies are to S1 (Fig. 6e). DTP does not induce production of detectable antibodies to S1 or holotoxin (Fig. 6e). As expected, mRNA-DTP-10 induces strong antibody responses to both S1 and holotoxin (Fig. 6e). Collectively these data show that mRNA-DTP10 can induce PT neutralizing antibodies.

## Discussion

The major goal of this study was to evaluate the mRNA platform as an approach for developing a multivalent diphtheria, tetanus, and pertussis vaccine. Diphtheria and tetanus vaccines have excellent track records at preventing disease; however, pertussis vaccines have a sorted past, where the most protective vaccine (DTP whole cell pertussis) was highly reactogenic and replaced by a well-tolerated acellular DTaP vaccine with demonstrated short-lived immunity^5,60–62,66,67^. In this study, we investigated if pertussis, diphtheria and tetanus antigens created as an mRNA vaccine could be immunogenic and provide protection against disease in a mouse model of *B. pertussis* infection. We observed antibody (Fig. 1) as well as T cell immunogenicity (Fig. 2). Additional antigen mRNA constructs (RTX, TCFA, SPHB1) (Fig. 3b) were combined with the pertussis antigen mRNA constructs PT, FHA, FIM to formulate resulting mRNA-P6 vaccine (Fig. 3c, Table 2). This vaccine induced antibodies that recognized the bacterium (Fig. 3d), PT (Fig. 3e) and significantly reduced bacterial burden and IL-6 in the lungs of mice at day 3 post challenge (Fig. 3fg). mRNA-DTP-10 vaccine induced production of antibodies that bind whole *B. pertussis* bacterium, and were more balanced as Th1 and Th2 type antibody isotypes, in contrast to DTaP immunized mice that elicited primarily Th2 type antibodies. (Fig. 4c–e). Addition of DT and TT mRNA constructs did not interfere with antibody titers to pertussis antigens (Fig. 4f). Mice immunized with mRNA-DTP-10 had significantly reduced bacterial burden of both UT25 and D420 pertussis strains (Fig. 5b, d) and reduction of leukocytosis in the lung (Fig. 5c, e) during a 7 day time course study. Furthermore, mice immunized with mRNA-DTP-10 were protected from PT challenge and showed normal white blood cell, neutrophil, and lymphocyte levels in blood (Fig. 6b–d).

The “pertussis problem” has seemed to be addressed twice in the past century since the causative agent, *B. pertussis* was isolated^68^. Diphtheria and tetanus diseases are fully mediated by single toxins. Toxoid DT and TT antigens, adsorbed to alum adjuvant induce protective antibodies. However, *B. pertussis* expresses not one toxin, but a full repertoire including: the namesake pertussis toxin, adenylate cyclase toxin (ACT), type III secretion system effectors, tracheal cytotoxin, dermonecrotic toxin, endotoxin, etc.^69^. Neutralization of pertussis toxin is attributed to decreased disease and increased survival against infection in infants^65^. However, DTaP and Tdap vaccines do not fully protect against colonization or transmission and therefore, pertussis remains a “vaccine preventable” disease that continues to circulate^14^. The DTP whole cell vaccine is known to induce Th1 responses including cell mediated immunity and opsonizing antibodies. The DTP also induces neutrophil recruitment via Th17 responses^17,70^. It is difficult to envision such adjuvants being used in infants or children due to the levels of inflammation they would cause at administration. mRNA vaccines have emerged from theoretical to pre-clinical and now are widely used to provide immunity to COVID-19. mRNA vaccines such as Spikevax/mRNA-1273 or Comirnaty/BNT162b2 encode one or two antigens and provide exceptional protection against SARS-CoV-2. Furthermore, mRNA vaccines are now recommended for children in addition to adults. Here in our studies, we have shown that mRNA vaccines can express up to 10 antigens and provide protection against *B. pertussis* in murine challenge studies. The pertussis problem is connected to length of protection and one caveat of our work is that we have not investigated how long mRNA pertussis immunity will last. However, we have measured memory T cell responses induced by mRNA-DTP vaccines (Fig. 2). There are examples of *B. pertussis* challenge studies in 6 month or older mice^25,71,72^. However, it is likely that human clinical studies are necessary to determine duration of protection from mRNA immunization.

*B. pertussis* is an obligate human pathogen that is transmitted by respiratory aerosols. In our studies here we used experimental challenge with in vitro culture of *B. pertussis* and intranasal administration using a liquid inoculum. The intranasal instillation model does not address the variable of host-to-host transmission. It has been hypothesized that the infectious dose in humans and baboons is likely a very low number of bacteria. While we do see that the mRNA pertussis vaccines were able to facilitate clearance of *B. pertussis* bacteria from the lower respiratory track of mice, we don’t have data to on how well these vaccines would protect against transmission. The baboon model of transmission would likely be the ideal model to evaluate this variable.

The DTP whole cell vaccine contains thousands of protein antigens along with endotoxin whereas acellular vaccines contain one (PT) to four (PT, FHA, PRN, FIM) antigens^1^. Another important consideration when comparing DTP and DTaP vaccines is the massive difference in total antigen. DTP whole cell vaccine delivers approximately 3 mg of protein antigen whereas DTaP contains roughly 100 µg of antigen. It is very difficult to compare these vaccines and their protection levels despite there are many attempts in the literature comparing these vaccines. Using proteomic analysis it is known that there are approximately 20 immunodominant antigens of *B. pertussis* as observed in whole cell or outer membrane vesicle (OmPv) immunized mice^12^. Therefore, it is likely that including additional antigens that will induce neutralizing or bacterial surface binding antibodies could improve protection. With the mRNA vaccines, we explored the inclusion of the repeats in toxin (RTX) antigen of ACT, TCFA, BRKA, and SPHB1 antigens. Adenylate cyclase toxin is an important virulence factor that is required for lethality of *B. pertussis* in neonate mice and it has long been known as a protective antigen^20,73^. A study from our lab showed that the addition of RTX, to 1/80th the human dose of DTaP increased *B. pertussis* clearance in the respiratory tract, reduced inflammatory cytokines in the lung, and increased the production of anti-*B. pertussis* IgG when compared to mice immunized with DTaP alone^51^. In the mRNA-DTP-10, RTX was included because it is reasonable to speculate that neutralization of ACT through RTX binding antibodies will enhance innate immune responses due to blocking the effects of the ACT^20^. It has been shown that anti-BrkA antibodies added to human serum improve bactericidal activity against *B. pertussis* by neutralizing BrkA, supporting idea of BrkA as a potential antigen candidate^74^. It has been reported that genetic deletion of *sphb1* gene attenuates *B. pertussis* pathogenesis in mice suggesting it is an important factor^75^; adding Sphb1 antigen to DTaP has been observed to increase protection^76,77^. Raeven et al. demonstrated that vaccination with outer membrane vesicles (OMV) containing BrkA and Sphb1 results in protection in murine challenge studies^78^. Here in our studies, we included RTX, TCFA, BRKA, and SPHB1 antigens into the mRNA-DTP-10 formulation and observed robust protection (Fig. 5).

One challenge that is difficult to normalize is mRNA dosage versus whole cell or acellular vaccines. Whole cell vaccines were normalized per units of what was protective in the mouse intracranial challenge model^79,80^. Current acellular vaccines used today were formulated based on the early acellular Japanese pertussis vaccines^2,81,82^. For mRNA, we do not know the exact amount of protein antigen that they produce per dose in the muscle. There are several reasons this is difficult to determine. However, we performed pilot studies from 1 to 10 µg and our mRNA combo vaccines were evaluated at 10 µg dose. There is more work that needs to be done in order to make proper comparisons between mRNA, acellular, and whole cell versions of pertussis vaccines. Given the fact that mRNA-1273 was used in prime/boost administration in humans at 100 µg, and the fact that formulation only has 1 antigen, it is possible that multivalent vaccines, may need to be studied at higher doses to accomplish sufficient responses to a multifaceted pathogen such at *B. pertussis*. Furthermore, the efficacy of the diphtheria and tetanus antigens would need to be evaluated as well. In this study, we have demonstrated proof of concept that multivalent mRNA vaccines for bacterial pathogens can be robustly immunogenic in rodents and protect against *B. pertussis* challenge. We propose to further characterize and optimize the mRNA encoded antigens to better understand the structural moieties contributing to the protection observed in the mouse challenge model. In subsequent follow-up studies, we also plan to investigate whether mRNA vaccine could address Th2 polarization in an acellular pertussis primed population and reduce bacterial colonization using the baboon model for pertussis.

## Methods

### mRNA antigen design

Mammalian signal peptides of either IgG kappa (METPAQLLFLLLLWLPDTTG) or bovine prolactin (MDSKGSSQKGSRLLLLLVVSNLLLPQGVVG) were used for all pertussis and tetanus antigens. A native signal sequence from *Corynebacterium diphtheriae* was maintained in the diphtheria antigen construct. In cases where the native bacterial antigen contained a putative mammalian signal N-linked glycosylation motif (eg. NXT/S), predictions via NetNGlyc Server, mutations were introduced at either the site of the native N or T/S to prevent non-native N-linked glycosylation from occurring^83^. In some cases, native antigens were additionally truncated, detoxified, or modified as described in Table 1 in order to optimize expression in mammalian cells^84^.

### Confirmation of protein expression in vitro using JESS system

15 ug mRNA constructs were transfected into 15 mls of Expi293F cells (1 × 10^6^ cells/ml) in Expi293 medium according to TransIT-mRNA transfection kit (Mirus) instructions. Cells were incubated with shaking at 37 °C + 5% CO_2_ for 48 h. Cells were centrifuged and the supernatant was collected. Samples were concentrated using Amicon filters of appropriate molecular weight cut off and centrifuged at 3000 *g* for 10 min. Supernatants were diluted with 0.1X sample buffer (cat SM-W004), denatured with 400 mM DTT (PS-ST01EZ-8) and boiled for 5 min. Primary antibodies (Supplemental Table 1) were diluted with antibody diluent 2 (042-203) (All buffers from Biotechne). Samples were loaded onto Jess plate and ran on automated Jess system according to manufacturer’s instructions).

### Vaccination model for Luminex antibody analysis and T cell activation

8-week-old C57BL/6J mice (Charles River) were immunized and boosted 4 weeks later with Alum, DTaP (Daptacel, Sanofi), mRNA-DTP-6, or a control mRNA formulated vaccine containing a non-coding mRNA. Sera and spleens were collected 1-month post boost and sera was evaluated for antibody binding by Luminex using beads coated with pertussis, diphtheria and tetanus antigen proteins and spleens were evaluated for T cell activation and immunophenotyping by flow cytometry. Serial dilutions of sera were prepared and incubated with Luminex using beads coated with native antigen purified from *Bordetella pertussis* (FHA, FIM2/3, PRN), detoxified PTX, DT or TT toxoid proteins for 2 h, shaking at 1000 rpm. Beads were washed with PBS 0.1% BSA 0.05% Tween-20, then incubated with secondary antibody (Goat anti-mouse PE), shaking for 1 hr at 1000 rpm. Beads were washed again and mean fluorescence intensity of bead binding was determined by Luminex.

### Flow cytometry-based T cell analysis

Splenocytes were homogenized using a Miltenyi gentleMACS on the spleen_01 setting, and RBC’s were removed using ACK lysis buffer and washed with HBSS and 2% FBS, before cells were adjusted to 1 × 10^7^ cells/ml. 2 × 10^6^ cells were stained with zombie violet live dead stain for 10 min in the dark, followed by treatment with FC Block for 10 min. Cells were then stained with extracellular surface markers for immunophenotyping T cells (CD4-PE, Cat. #116006, (1:800); TCR-β -PerCP-Cy5.5, Cat. #109228, (1:300); CD44-PE-Cy7, Cat. #103030, (1:300); CXCR3-APC, Cat. #126511, (1:100); CD8-APC-Cy7, Cat. #100714, (1:300); CD62L-BV605, Cat. #104438, (1:400); CD3-BV711, Cat. #100241, (1:100); CD45-AF700, Cat. #103128, (1:300)). All antibodies used for flow cytometry staining are BioLegend. For intracellular cytokine analysis 2 ×10^6^ cells were stimulated with PMA (50 ng/ml) /Ionomycin 500 ng/ml) and treated with golgiStop™ (1 ug/ml) for 5 h at 37 °C + 5% CO_2_. Cells were then washed and treated with live dead and FC block, and extracellular markers (CD3-FITC, Cat. #100306, (1:300); TCR-β -PerCP-Cy5.5, Cat. #109228, (1:300); CD4-APC, Cat. #116014, (1:300); CD8-APC-Cy7, Cat. #100714, (1:300); CD45-AF700, Cat. #103128, (1:300)) as done for immunophenotyping the cells. Then cells were washed with staining buffer (PBS and 2% FBS) and permeabilized with permwash (BD) and fixed for 30 min at 4 °C with cytofix buffer (BD) before further washes with perm wash. Cells were then stained with antibodies against intracellular cytokines (IL-13-PE, Cat. #159403, 1:50; IFNγ-BV711, Cat. #505836, 1:50) for 45 min at 40 °C, then washed and data was acquired on an Attune NxT (Thermo Fisher). All antibodies and live dead stain dye were purchased from Biolegend. Gating strategies are shown in supplemental Fig. 3.

### Vaccine compositions

INFANRIX (GSK) or DAPTACEL (Sanofi Pasteur) human vaccines (DTaP), and the National Institute for Biological Standards and Control whole-cell pertussis vaccine (NIBSC code 94/532) or Serum Institute of India Triple Antigen DTP were diluted in 1X endotoxin free PBS and administered at 1/20th the human dose for all studies. mRNA vaccines encoding pertussis, diphtheria and tetanus antigens were synthesized in vitro by T7 polymerase RNA transcription reaction using N1-methyl-pseudouridine. Purified mRNAs were then encapsulated in lipid nanoparticles using an ethanol drop nanoprecipitation method, as described previously^28,85,86^. For single antigen vaccines, individual mRNA were formulated into individual LNPs. Combinational vaccines were formulated by adding each mRNA at a 1:1 mass ratio. Both types of formulations resulted in a homogenous mRNA-LNP formulation. mRNA vaccine dose was 10 µg total mRNA for all studies, except where noted.

### *B. pertussis* strain and growth conditions

*B. pertussis* strain UT25Sm1 (UT25Sm1 NCBI Reference Sequence: NZ_CP015771.1), which was isolated in Texas from a nasopharyngeal swab in 1977, was used for murine challenge in all experiments^87^. UT25Sm1 was cultured on Bordet Gengou agar (Difco™, BD, Cat. #297876) with 15% defibrinated sheep’s blood (Hemostat Laboratories) with streptomycin 100 µg/mL (Gibco™, Cat. #11860038). *B. pertussis* isolate D420 (European Nucleotide Archive the accession number LN849008) was kindly provided by Drs. Maria L. Tondella and Michael Weigand (Center for Disease Control, Atlanta, Georgia)^55,88^. D420 was cultured in the same manner as UT25Sm1 with the exception that no antibiotics were added to the Bordet Gengou agar. Both UT25Sm1 and D420 inoculated plates were incubated in 36 °C incubators for 48 h then transferred to modified Stainer-Scholte liquid medium (SSM) supplemented with L-proline and SSM supplement^89^. Liquid cultures were incubated for 24 h at 36 °C, with shaking at 180 rpm until reaching an OD_600_ of ~0.6, at which time cultures were diluted for the challenge dose to an OD_600_ of 0.245 (equivalent to 1 × 10^9^ CFU/mL) using a Beckman Coulter™ DU 530 UV vis spectrophotometer.

### Vaccine administration, *B. pertussis* challenge, and day 3 sample collection

Immunization studies used two mouse strains C57BL/6J (The Jackson Laboratory) and BALB/c (Charles River Laboratories; inbred; strain code 028). C57BL/6 J mice were used for study shown in Figs. 1, 2, 3 and BALB/c mice were used studies shown in Figs. 4, 5, 6). The female mice were aged four weeks upon arrival and randomly assigned 5 mice per cages by veterinary staff. At five weeks cages were assigned a group by randomly labeling the cage with the vaccine group and the mice were immunized intramuscularly with 50 µL of vaccine or vehicle control. Mice were boosted with the same vaccine formulations four weeks after priming.

At six weeks post initial vaccination, mice were anesthetized and intranasally challenged with a total of 2 × 10^7^ CFUs of *B. pertussis* (10 µL of culture per nostril). Mice were anesthetized by intraperitoneal injection of 200 μl of ketamine (6.7 mg/ml) and xylazine (1.3 mg/ml) in 0.9% saline. At 3 days post-challenge, mice were euthanized by pentobarbital injection (390 mg/kg, Euthasol, Patterson Veterinary) and blood was collected via cardiac puncture. A portion of collected blood (250 μL) was transferred to a BD microtainer tubes with K_2_EDTA and complete blood counts were determined using an IDEXX Procyte. Serum was separated by centrifugation (15,000 × *g* for 3 min) via a BD Microtainer blood collector (BD, Cat# 365974) and stored at −80 °C until analysis. The trachea and lungs were removed and suspended in 1 mL of sterile PBS inside a GentleMACS C tube (Miltenyi, Cat# 130096334) and homogenized using a GentleMACS Octo Dissociator with Heaters (Miltenyi) using the m_lung_02 setting. Lung and trachea homogenate were used to determine colony forming units (CFUs) via serial dilutions. A sample of lung homogenate was centrifuged a 14,000 × *g* for 4 min and the supernatants were stored in −80 °C until cytokine and antibody analyses. Serial dilutions were done in sterile 1× PBS and then plated on BG containing streptomycin (100 μg/mL) to ensure that only UT25 *B. pertussis* was cultured. BG plates containing cephalexin (40 µg/mL) were used for samples from mice challenged with D420. To lower the limit of CFU detection for study 3, a cell-spreader was used with a 100uL aliquot of the lung/trachea homogenate.

### Serological analysis of *B. pertussis* specific antibodies

Serological responses to vaccination were quantified by ELISA. High-binding microtiter plates were coated with PT (50 ng/well) (PT#180, LIST Biologicals), as described in Boehm et al.^20^. Plates were also coated with RTX-751 (GenScript), PRN (GenScript), FHA (Native antigen BP-FHA-100), diphtheria toxin (Abcam 188505), tetanus toxin (Enzo ALX630108) at 50 ng/well. For serological responses to *B. pertussis*, UT25Sm1 and D420 were cultured to an OD_600_ of ~0.6 and diluted down to an OD_600_ of 0.245 and microtiter plates were coated with 50 µL of diluted solution per well. After coating overnight at 4 ^o^C, the plates were washed three times with PBS + 0.05% v/v Tween 20 (Fisher Scientific) (PBS-T) and blocked with 5% non-fat dry (NFD) milk in PBS-T overnight at 4 °C. Blocked plates were washed with PBS-T and then serum (1:50) were prepared in 5% NFD milk in PBS-T. All samples were serially diluted (1:2). After 2 h incubation at 37 °C, plates were washed and incubated with either goat anti-mouse-total IgG alkaline-phosphatase conjugated antibody (Southern Biotech, Cat. #1030-04), goat anti-mouse IgG2a alkaline-phosphatase conjugated antibody (Southern Biotech, Cat. #1081-04), goat anti-mouse IgG2b alkaline-phosphatase conjugated antibody (Southern Biotech, Cat. #1091-04), or goat anti-mouse IgG1 (Southern Biotech, Cat.#1071-04) for 1 h at 37 °C (all antibodies diluted 1:2000 in 5% NFD milk in PBS-T). Plates were then washed and incubated with Pierce *p*-Nitrophenyl Phosphate (PNPP) (Thermo Fisher Scientific, Cat. # 34047) following the manufacturer’s instructions. The absorbance of the plates was read at OD_405_ using a Synergy H1 plate reader (BioTek). Positive antibody titers were determined as any values above the baseline (set at two times the average of blanks).

### Lung homogenate cytokine analysis

To quantify inflammatory cytokines at the site of infection, lung homogenate supernatants were prepared as suggested by the kit manufacturer and diluted 1:2 in provided buffer. Quantitative analysis of cytokines was performed using: V-PLEX mouse IL-6 kit (MSD, K152QXD-1) [study 1], ProcartaPlex Mouse IL-6 Simplex (Invitrogen EPX01A-20603-901) [study 2] and Mouse Magnetic Luminex Assay Premixed multiplex (R&D Systems) [study 3], per the manufacturer’s instructions. Samples were run on a MAGPIX instrument (Luminex Corporation). Seven standards (in duplicates) were used for every kit. 50 µL of each sample was used for analysis and bead counts below 35 were invalidated.

### Pertussis toxin challenge model

BALB/c mice were immunized in the same manner and timeframe as listed above. 2 weeks post-boost, mice were challenged with 2 µg PT (List Biological, #180). In short, 50 µg lyophilized PT was reconstituted in 500 µL 1x endotoxin free PBS. Per mouse challenged, 20 µL of the PT solution was diluted in 180 µL 1x endotoxin free PBS and administered via IP injection. At day 3 post-challenge, mice were euthanized with pentobarbital and a cardiac puncture was performed to collect blood. 250 µL of the blood was added to a BD microtainer tubes with K_2_EDTA (BD, Cat#365974) and complete blood counts were determined using an IDEXX Procyte.

### Statistical analysis

Statistical analyses were performed using Prism version 10 (GraphPad). Comparisons between three or more groups that followed a normal distribution were analyzed by one-way analysis of variance (ANOVA) followed by a Tukey’s multiple comparison test. Statistical analysis for non-parametrical data was performed using the Kruskal-Wallis test with a Dunn’s post-hoc test. Differences between two groups was determined by Student’s *t* test. *P* values below 0.05 were considered significant. Correlations between two parameters were calculated via linear regression analysis of log-transformed data. For T-cell analysis, a Mann-Whitney two-tailed with 95% confidence interval test was used to determine statistical difference from mRNA-DTP-6

### Reporting summary

Further information on research design is available in the Nature Research Reporting Summary linked to this article.

### Supplementary information

Supplementary Information

Reporting Summary

## Data Availability

Data for all figures are available upon reasonable request to the corresponding author.
