# Supplementary material for: Multivalent mRNA-DTP vaccines are immunogenic and provide protection from Bordetella pertussis challenge in mice
Source: NPJ Vaccines. 2024 Jun 10;9:103. doi: 10.1038/s41541-024-00890-4 (PMC11164898; doi:10.1038/s41541-024-00890-4)
Supplement: Supplementary file 1 — Supplementary Information [file 41541_2024_890_MOESM1_ESM.pdf]

Supplemental figures:

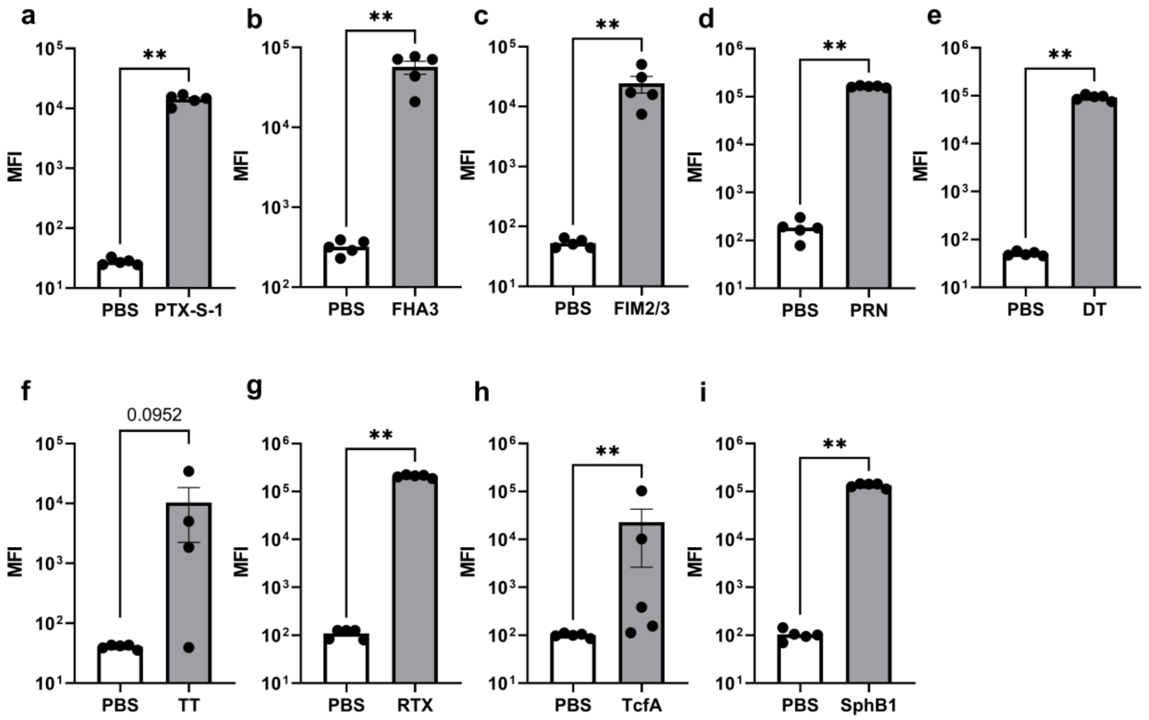

Supplemental Figure 1. mRNA encoded pertussis, diphtheria, and tetanus toxoid antigens are immunogenic in mice. 8-week-old BALB/c mice (Charles River) were immunized IM twice at monthly intervals with 2 ug of formulated LNP-mRNA encoding either PTX-1, FHA3, FIM2/3, PRN, DT, TT, RTX, TCFA, or SphB1 or administered PBS IM, and sera was collected one-month post-boost. Luminex beads were coated with native antigen purified from *Bordetella pertussis* (FHA, FIM2/3, PRN), detoxified PTX, DT or TT toxoid, or recombinant versions of RTX, TCFA and SphB1 that were expressed in *E. coli*. Sera were incubated with beads and antibody binding was evaluated by mean fluorescence intensity (MFI) by Luminex. A Student's t-test was performed to compared differences in fluorescence between PBS control sera and single antigen vaccinated sera for each vaccine antigen; *p<0.05, ***p<0.005, ****p<0.001 indicate differences from PBS control. Error bars are ± SD.

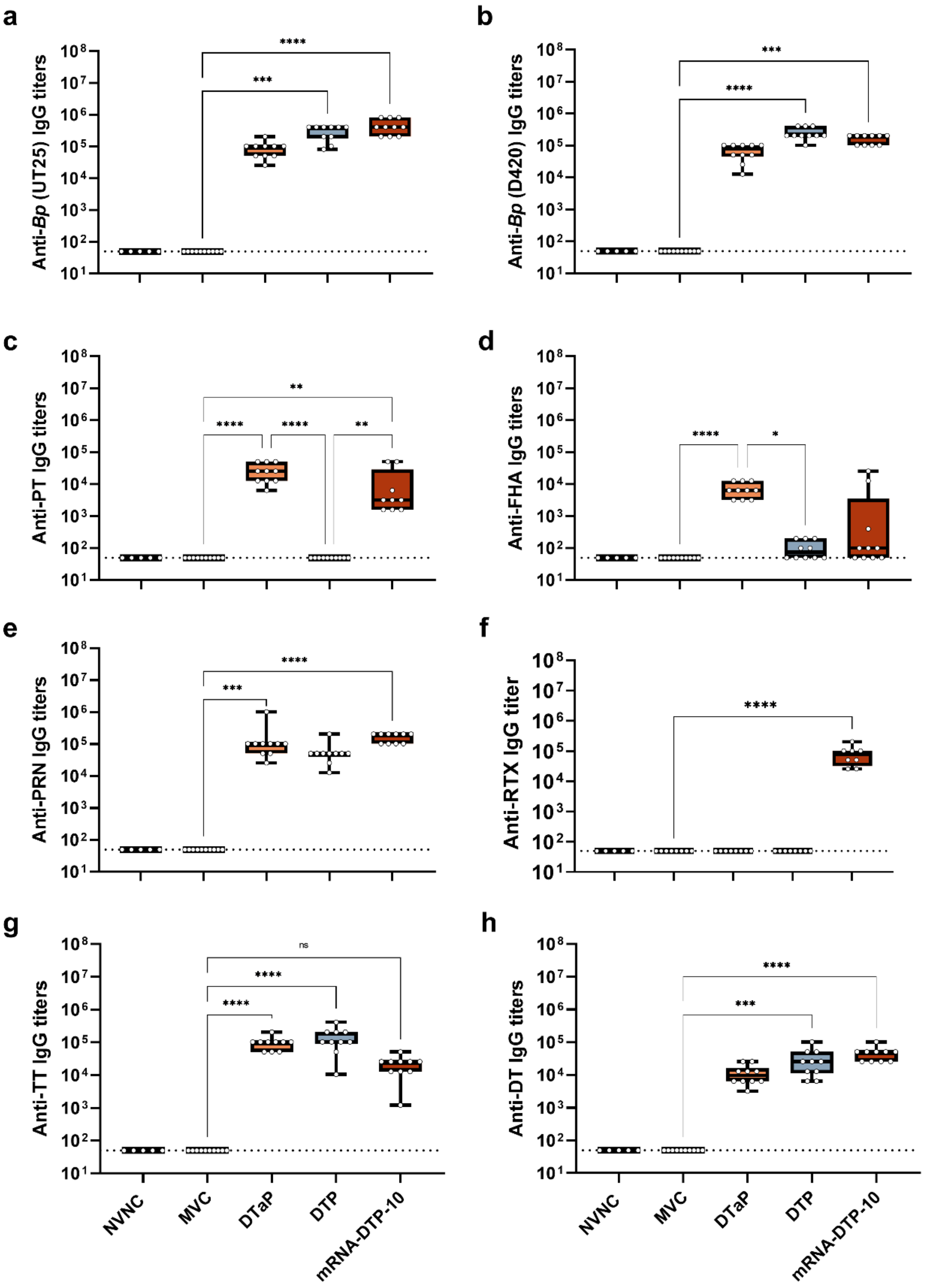
a
Anti-Bp (UT25) IgG titers
b
Anti-Bp (D420) IgG titers
c
Anti-PT IgG titers
d
Anti-FHA IgG titers
e
Anti-PRN IgG titers
f
Anti-RTX IgG titer
g
Anti-TT IgG titers
h
Anti-DT IgG titers
ns
NVNC
MVC
DTaP
DTP
mRNA-DTP-10

Supplemental Figure 2. A multivalent mRNA-DTP is immunogenic in mice. Post-boost serological IgG titers to pertussis, diphtheriae, and tetanus antigens. IgG antibodies to *B. pertussis* strains UT25 (a), D420 (b), PT (c), FHA (d), PRN (e), RTX (f), TT (g), DT (h). Kruskal-Wallis with Dunn's post-hoc test was performed to calculate differences. *$p<0.05$, ***$p<0.005$, ****$p<0.001$ indicate differences from MVC. Box and whisker plots display minimum to maximum values with all data points. NVNC= non-vaccinated, non-challenged; MV=mock-vaccinated; n=10 per treatment group, except for NVNC n=5. Dotted lines indicate lowest limit of detection. Error bars are ± SEM.

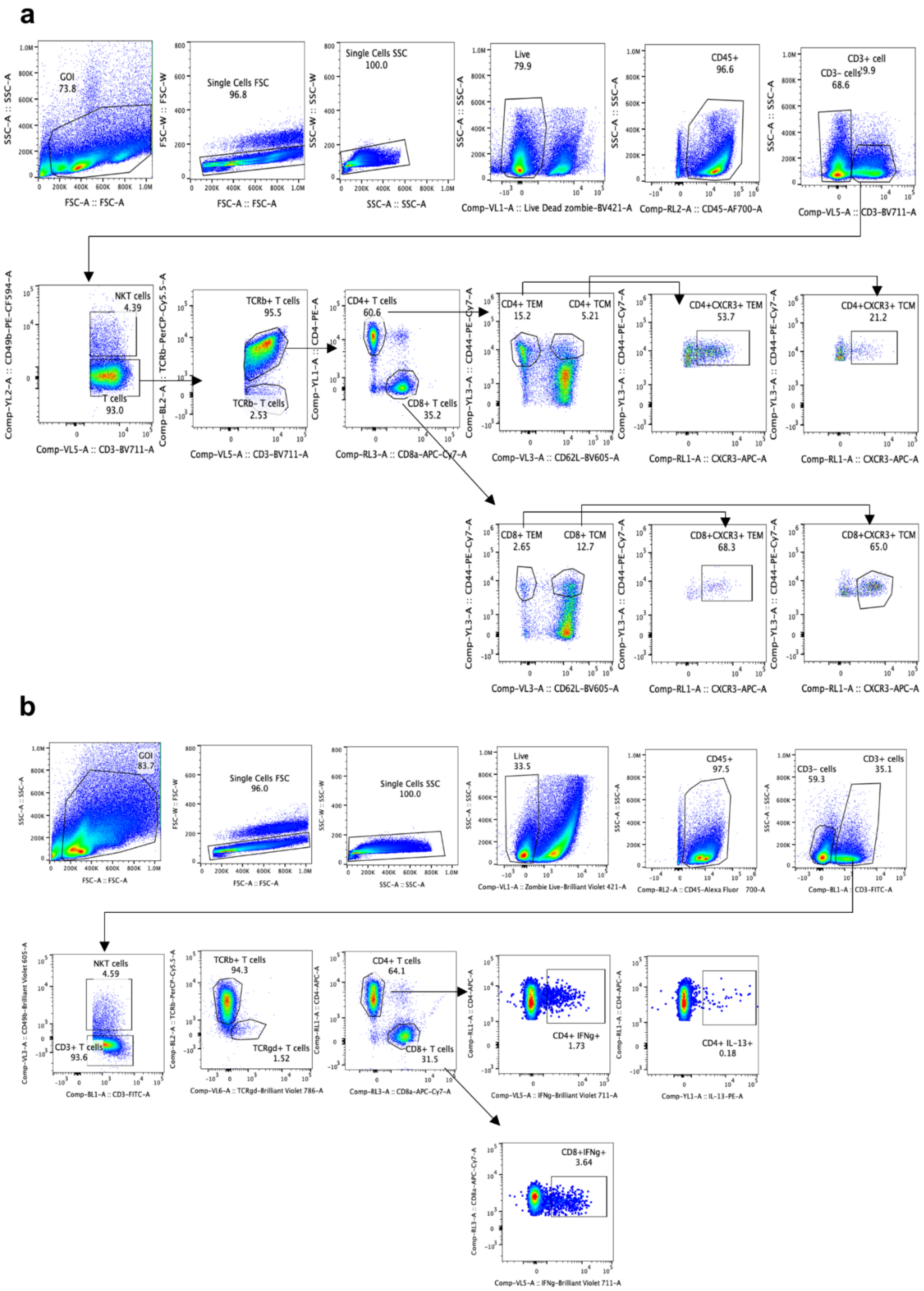
a
GOI
73.8
SSC-A :: SSC-A
FSC-A :: FSC-A
Single Cells FSC
96.8
FSC-W :: FSC-W
Single Cells SSC
100.0
SSC-W :: SSC-W
SSC-A :: SSC-A
Live
79.9
Comp-VL1-A :: Live Dead zombie-BV421-A
CD45+
96.6
Comp-RL2-A :: CD45-AF700-A
CD3+ cell
CD3- cells
79.9
68.6
Comp-VL5-A :: CD3-BV711-A
NKT cells
4.39
T cells
93.0
Comp-YL2-A :: CD49b-PE-CF594-A
Comp-VL5-A :: CD3-BV711-A
TCRb+ T cells
95.5
TCRb- T cells
2.53
Comp-BL2-A :: TCRb-PerCP-Cy5.5-A
Comp-VL5-A :: CD3-BV711-A
CD4+ T cells
60.6
CD8+ T cells
35.2
Comp-YL1-A :: CD4-PE-A
Comp-RL3-A :: CD8a-APC-Cy7-A
CD4+ TEM
15.2
CD4+ TCM
5.21
Comp-YL3-A :: CD44-PE-Cy7-A
Comp-VL3-A :: CD62L-BV605-A
CD4+CXCR3+ TEM
53.7
CD4+CXCR3+ TCM
21.2
Comp-RL1-A :: CXCR3-APC-A
CD8+ TEM
2.65
CD8+ TCM
12.7
CD8+CXCR3+ TEM
68.3
CD8+CXCR3+ TCM
65.0
b
GOI
83.7
SSC-A :: SSC-A
FSC-A :: FSC-A
Single Cells FSC
96.0
FSC-W :: FSC-W
Single Cells SSC
100.0
SSC-W :: SSC-W
SSC-A :: SSC-A
Live
33.5
Comp-VL1-A :: Zombie Live-Brilliant Violet 421-A
CD45+
97.5
Comp-RL2-A :: CD45-Alexa Fluor 700-A
CD3- cells
59.3
CD3+ cells
35.1
Comp-BL1-A :: CD3-FITC-A
NKT cells
4.59
CD3+ T cells
93.6
Comp-VL3-A :: CD49b-Brilliant Violet 605-A
TCRb+ T cells
94.3
TCRgd+ T cells
1.52
Comp-BL2-A :: TCRb-PerCP-Cy5.5-A
Comp-VL6-A :: TCRgd-Brilliant Violet 786-A
CD4+ T cells
64.1
CD8+ T cells
31.5
Comp-RL1-A :: CD4-APC-A
Comp-RL3-A :: CD8a-APC-Cy7-A
CD4+ IFNg+
1.73
Comp-VL5-A :: IFNg-Brilliant Violet 711-A
CD4+ IL-13+
0.18
Comp-YL1-A :: IL-13-PE-A
CD8+IFNg+
3.64
Comp-RL3-A :: CD8a-APC-Cy7-A
Comp-VL5-A :: IFNg-Brilliant Violet 711-A

Supplemental Figure 3. Gating strategies for T cell flow cytometry analysis. (a) Gating strategy for CD4 and CD8 T cell effector and central memory analysis from vaccinated spleen. (b) Gating strategy for CD4 and CD8 T cells from vaccinated spleen stained for intracellular cytokine production.

Supplementary Table 1

Antigen	Primary Antibody	Primary Antibody dilution	Source/catalogue #
PTX-S1	1B7 mAb (mouse)	1:100	NIBSC/ 99/506
FHA3	2E9 mAb (mouse)	1:200	NIBSC/ 99/572
Fim2/3	Fim2 specific;0603 rabbit pAb	1:150	MyBioSource/MBS1490603
Fim2/3	Fim3 specific; 9657 rabbit pAb	1:100	MyBioSource/MBS1499657
PRN	Anti-rPRN rabbit pAb	1:100	Alpha Diagnostics Intl/PRN11-A
DT	Anti-DT toxoid goat pAb	1:50	Thermo / PA1-7212
TT	Anti-Frag C Rabbit pAb	1:100	Rockland/ 100-401-894
