# Supplementary material for: Multivalent mRNA-DTP vaccines are immunogenic and provide protection from Bordetella pertussis challenge in mice
Source: NPJ Vaccines. 2024 Jun 10;9:103. doi: 10.1038/s41541-024-00890-4 (PMC11164898; doi:10.1038/s41541-024-00890-4)
Supplement: Supplementary file 2 — Reporting Summary [file 41541_2024_890_MOESM2_ESM.pdf]

nature portfolio

Corresponding author(s): DAMRON

Last updated by author(s): 2024-0123

Reporting Summary

Nature Portfolio wishes to improve the reproducibility of the work that we publish. This form provides structure for consistency and transparency in reporting. For further information on Nature Portfolio policies, see our Editorial Policies and the Editorial Policy Checklist.

Statistics

For all statistical analyses, confirm that the following items are present in the figure legend, table legend, main text, or Methods section.

n/a	Confirmed	
☐	☒	The exact sample size (*n*) for each experimental group/condition, given as a discrete number and unit of measurement
☐	☒	A statement on whether measurements were taken from distinct samples or whether the same sample was measured repeatedly
☐	☒	The statistical test(s) used AND whether they are one- or two-sided<br>*Only common tests should be described solely by name; describe more complex techniques in the Methods section.*
☐	☒	A description of all covariates tested
☐	☒	A description of any assumptions or corrections, such as tests of normality and adjustment for multiple comparisons
☐	☒	A full description of the statistical parameters including central tendency (e.g. means) or other basic estimates (e.g. regression coefficient) AND variation (e.g. standard deviation) or associated estimates of uncertainty (e.g. confidence intervals)
☐	☒	For null hypothesis testing, the test statistic (e.g. *F*, *t*, *r*) with confidence intervals, effect sizes, degrees of freedom and *P* value noted<br>*Give P values as exact values whenever suitable.*
☒	☐	For Bayesian analysis, information on the choice of priors and Markov chain Monte Carlo settings
☒	☐	For hierarchical and complex designs, identification of the appropriate level for tests and full reporting of outcomes
☒	☐	Estimates of effect sizes (e.g. Cohen's *d*, Pearson's *r*), indicating how they were calculated

Our web collection on statistics for biologists contains articles on many of the points above.

Software and code

Policy information about availability of computer code

Data collection	no sofware used
Data analysis	Prism version 10 (GraphPad)

For manuscripts utilizing custom algorithms or software that are central to the research but not yet described in published literature, software must be made available to editors and reviewers. We strongly encourage code deposition in a community repository (e.g. GitHub). See the Nature Portfolio guidelines for submitting code & software for further information.

Data

Policy information about availability of data

All manuscripts must include a data availability statement. This statement should provide the following information, where applicable:

- Accession codes, unique identifiers, or web links for publicly available datasets
- A description of any restrictions on data availability
- For clinical datasets or third party data, please ensure that the statement adheres to our policy

none

Research involving human participants, their data, or biological material

Policy information about studies with human participants or human data. See also policy information about sex, gender (identity/presentation), and sexual orientation and race, ethnicity and racism.

Reporting on sex and gender	All experiments were performed with female mice. Comparisons to male mice were not performed.
Reporting on race, ethnicity, or other socially relevant groupings	not applicable
Population characteristics	not applicable
Recruitment	not applicable
Ethics oversight	oversight by WVU IACUC

Note that full information on the approval of the study protocol must also be provided in the manuscript.

Field-specific reporting

Please select the one below that is the best fit for your research. If you are not sure, read the appropriate sections before making your selection.

☒ Life sciences ☐ Behavioural & social sciences ☐ Ecological, evolutionary & environmental sciences

For a reference copy of the document with all sections, see nature.com/documents/nr-reporting-summary-flat.pdf

Life sciences study design

All studies must disclose on these points even when the disclosure is negative.

Sample size	*Describe how sample size was determined, detailing any statistical methods used to predetermine sample size OR if no sample-size calculation was performed, describe how sample sizes were chosen and provide a rationale for why these sample sizes are sufficient.*
Data exclusions	no data were excluded
Replication	experiments were repeated as described
Randomization	mice were ordered and then randomized into groups
Blinding	experimental groups were numbered and the vaccines identities were not know to the researchers until after the completion of the studies and stats were performed.

Reporting for specific materials, systems and methods

We require information from authors about some types of materials, experimental systems and methods used in many studies. Here, indicate whether each material, system or method listed is relevant to your study. If you are not sure if a list item applies to your research, read the appropriate section before selecting a response.

Materials & experimental systems

n/a	Involved in the study	
☐	☒	Antibodies
☐	☒	Eukaryotic cell lines
☒	☐	Palaeontology and archaeology
☐	☒	Animals and other organisms
☒	☐	Clinical data
☒	☐	Dual use research of concern
☒	☐	Plants

Methods

n/a	Involved in the study	
☒	☐	ChIP-seq
☐	☒	Flow cytometry
☒	☐	MRI-based neuroimaging

Antibodies

Antibodies used	PTX-S1 1B7 mAb (mouse) source NIBSC/ 99/506<br>FHA3 2E9 mAb (mouse) source NIBSC/ 99/572<br>Fim2/3 Fim2 specific;0603 rabbit pAb MyBioSource/MBS1490603

	Fim2/3 Fim3 specific; 9657 rabbit pAb MyBioSource/MBS1499657<br>PRN Anti-rPRN rabbit pAb Alpha Diagnostics Intl/PRN11-A<br>DT Anti-DT toxoid goat Thermo / PA1-7212<br>TT Anti-Frag C Rabbit pAb Rockland/ 100-401-894
Validation	These antibodies were validated for western blot by testing them for specificity to purified proteins as well as against non-specific binding to HEK293T cells and supernatant for lack of cross reactivity

Eukaryotic cell lines

Policy information about cell lines and Sex and Gender in Research

Cell line source(s)	Expi293F
Authentication	cells were not authenticated; commercially purchased
Mycoplasma contamination	not verified
Commonly misidentified lines (See ICLAC register)	none

Animals and other research organisms

Policy information about studies involving animals; ARRIVE guidelines recommended for reporting animal research, and Sex and Gender in Research

Laboratory animals	Mus musculus C57BL/6J and BALBc
Wild animals	none
Reporting on sex	female mice only were used
Field-collected samples	none
Ethics oversight	WVU IACUC 1602000797_R1 and R2 Damron

Note that full information on the approval of the study protocol must also be provided in the manuscript.

Plants

Seed stocks	NA
Novel plant genotypes	NA
Authentication	NA

Flow Cytometry

Plots

Confirm that:

☒ The axis labels state the marker and fluorochrome used (e.g. CD4-FITC).

☒ The axis scales are clearly visible. Include numbers along axes only for bottom left plot of group (a 'group' is an analysis of identical markers).

☒ All plots are contour plots with outliers or pseudocolor plots.

☒ A numerical value for number of cells or percentage (with statistics) is provided.

Methodology

Sample preparation	Mouse splenocytes were homogenized using a Miltenyi gentleMACS on the spleen_01 setting, and RBC's were removed

Sample preparation	using ACK lysis buffer and washed with HBSS and 2% FBS, before cells were adjusted to 1 x107 cells/ml. 2 x106 cells were stained with zombie violet live dead stain for 10 minutes in the dark, followed by treatment with FC Block for 10 minutes.
Instrument	Attune NxT (Thermo Fisher
Software	Attune Cytometric Software
Cell population abundance	sorting was not performed
Gating strategy	gating strategy supplied in supplemental materials

☒ Tick this box to confirm that a figure exemplifying the gating strategy is provided in the Supplementary Information.
